# Self-Imaging Effect in Liquid-Filled Hollow-Core Capillary Waveguide for Sensing Applications

**DOI:** 10.3390/s20010135

**Published:** 2019-12-24

**Authors:** Yijian Huang, Shuhui Liu, Lichao Zhang, Yiping Wang, Ying Wang

**Affiliations:** 1 Guangdong and Hong Kong Joint Research Centre for Optical Fibre Sensors, College of Physics and Optoelectronic Engineering, Shenzhen University, Shenzhen 518060, China; huangyijian@email.szu.edu.cn (Y.H.); liushuhui@szu.edu.cn (S.L.); lczhang5354@szu.edu.cn (L.Z.); ypwang@szu.edu.cn (Y.W.); 2 Guangdong Laboratory of Artificial Intelligence and Digital Economy, Shenzhen University, Shenzhen 518060, China; 3 Key Laboratory of Optoelectronic Devices and Systems of Ministry of Education and Guangdong Province, Shenzhen University, Shenzhen 518060, China

**Keywords:** hollow-core capillary waveguide, self-imaging effect, temperature sensor

## Abstract

A high sensitivity fiber-optic sensor based on self-imaging effect in a hollow-core capillary waveguide (HCCW) is presented for sensing applications. The sensor is composed of a section of HCCW fusion spliced between single mode fibers (SMFs). The self-imaging effect in the HCCW is investigated with different fiber lengths and arc-fusion parameters. By infiltrating the hollow core with index matching liquids, the peak wavelength of the proposed device shifts towards longer wavelengths. The temperature and refractive index (RI) responses of the sensor are studied systematically. When temperature is increased from 25 °C to 75 °C, the temperature sensitivity of the device can be improved significantly with the infiltrated structure, and reaches −0.49 nm/°C, compared with that of the un-filled device, which is 9.8 pm/°C. For the RI response, the liquid-filled structure achieves sensitivity of 12,005 nm/RIU in the range between 1.448 and 1.450, slightly higher than the 11,920 nm/RIU achieved by the un-filled one. The proposed sensor exhibits the advantages of simple structure, high sensitivity and low cost, which may find potential applications in physical and chemical sensing.

## 1. Introduction

The development of new fiber-optic sensors has ignited widespread enthusiasm in the past few decades for the excellent characteristics exhibited by these sensors, such as high sensitivity, compact size, real-time sensing, corrosion resistance, and multiplexing capabilities. Fiber optic sensors are an ideal candidate for some special occasions such as temperature monitoring of flammable chemicals or explosive environments, because the fiber itself does not require electrical signals and can be monitored remotely. Numerous fiber-optic sensors based upon various operation mechanisms have been reported previously. For example, by means of employing fiber gratings [1], Mach-Zehnder interferometer [2], surface plasmon resonance [3], and photonic crystal fibers [4,5]. Unfortunately, the implementation of these structures often requires complex and time-consuming manufacturing processes, sophisticated processing equipment, or the use of expensive specialty fibers, which may greatly limit the mass production and economics of these devices. Optical devices fabricated using the self-image principle have exhibited several advantages such as simple structure, easy to manufacture, and fairly low cost, which has been adopted to a number of applications [6,7,8,9]. In 1975, Ulrich and Ankele described the self-imaging phenomenon in homogeneous planar optical waveguides [10]. Then, several types of multimode fiber (MMF) or coreless silica fiber (CSF) based sensors have been demonstrated such as refractive index (RI) sensor [11,12,13], temperature sensor [14], liquid level sensor [15,16], and displacement sensor [17].

Here, we demonstrate a unique fiber-optic sensor based on the self-imaging phenomenon in a hollow-core capillary waveguide (HCCW). The inherent air hole structure in HCCW makes this configuration ideal for integrating liquid materials such as thermo-optic materials, photosensitive materials [18] and magneto-optical materials [19] to create a variety of microfluidic fiber-optic devices. Regular variation of the transmission spectrum can be observed by simply changing the arc-fusion parameter or the length of the HCCW. Two samples with the same length of HCCW, one of which is infiltrated with index matching liquid and the other is not, are characterized for temperature and RI response measurements. Results show that the average temperature sensitivity can be improved to −0.49 nm/°C with liquid infiltration, compared to that of the un-infiltrated one (9.8 pm/°C) when temperature increases from 25 °C to 75 °C. And the RI sensitivity reaches 12,005 nm/RIU for the infiltrated sample in the range between 1.448 and 1.450. The proposed sensor exhibits good performances, such as simple structure, low cost, and high sensitivity, making it promising for wide applications in physical and chemical sensing.

## 2. Fabrication and Principle

**Figure 1:**
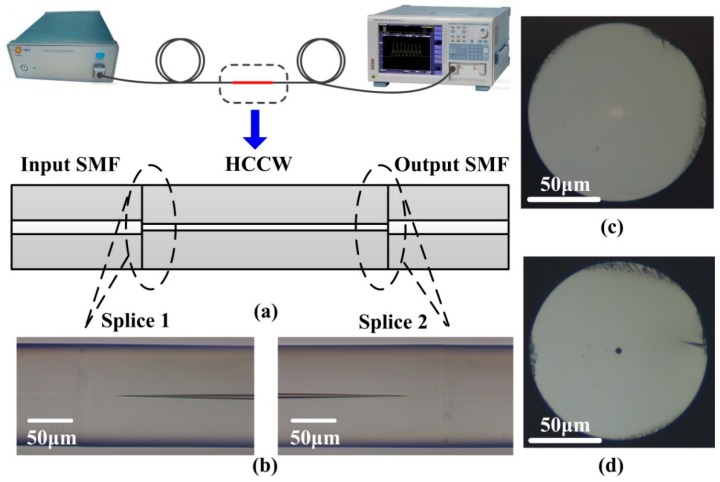
(**a**) Experimental setup for sensor transmission measurement, (**b**) microscope image of the splicing joints, (**c**,**d**), end face geometry of single mode fibers (SMF) and hollow-core capillary waveguide (HCCW), respectively.

A schematic of our proposed sensor is shown in Figure 1. In our experiment, the proposed sensor was fabricated by splicing a short HCCW between two standard single-mode fibers (SMFs). The HCCW (TSP005150, Polymicro Technologies, USA) fabricated from pure silica material has an outer diameter of 125 μm and an inner diameter of about 5 μm. A broadband light source (FL-ASE, FiberLake, Shenzhen, China) and an optical spectrum analyzer (OSA, AQ6370D, Yokogawa, Tokyo, Japan) were used to measure the transmission spectrum of the proposed sensor, as illustrated schematically in Figure 1a. Both ends of the HCCW are spliced to standard SMF using a commercial fusion splicer (FSM-80S, Fujikura, Tokyo, Japan), and the air holes of the HCCW were collapsed at both splicing points. Figure 1b shows the microscope (DM2700M, Leica, Germany) image of the two splicing joints. The end face geometry of the standard SMF and HCCW were shown in Figure 1c,d, respectively.

Moreover, there are two guiding mechanisms of light propagation in the proposed HCCW structure, namely, the well-known total internal reflection (TIR) and anti-resonant reflection guidance, which is well described by anti-resonant reflection optical waveguide (ARROW) [20]. At the first splicing joint of the proposed device, a portion of the input light that enters the air hole of HCCW directly may be guided through the ARROW mechanism according to the anti-resonant conditions, and the remaining input light that enters the silica wall of HCCW may propagate through TIR. However, the dominant guiding mechanism of the fabricated samples can be attributed to TIR because of the collapse regions induced in the two splicing joints during arc fusion process, as can be seen in Figure 1b, which may suppress the light power that enters the air hole directly. Due to the circular symmetry configuration of the HCCW, the optical propagation in the plane of the HCCW axis is simulated. In this plane, the HCCW can be considered to be two parallel multimode waveguides with thicknesses of 60 μm running along the HCCW axis, and the cladding is air. Also, the collapse of HCCW at both splicing joints are taken into account, with a no-core-region length of 38 μm and a hole-transition-region length of 130 μm. When the length of HCCW is appropriate, light propagated in the HCCW with specific wavelength could be coupled to the output SMF as a direct single image, i.e., self-imaging phenomenon in a multimode waveguide, and the light field of the image can be propagated in the SMF and forms a high transmittance peak in the transmission spectrum compared to other wavelengths. According to the self-imaging theory for high-contrast waveguides (air cladding) [6], the peak wavelength in the transmission spectrum for the first direct single image can be approximated using the formula below,

(1) $$\lambda =\frac{8nD^{2}}{L}$$

where *n*, *D* and *L* are the effective RI, wall-thickness and length of the HCCW, respectively.

**Figure 2:**
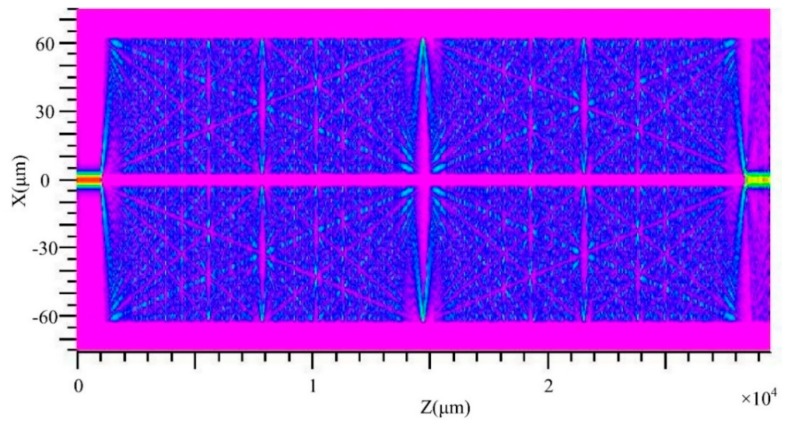
Self-imaging effect of the HCCW simulated by beam propagation method (BPM). Length of input/output SMF, 1 mm. Length of HCCW, 27.5 mm. Incident wavelength, 1551 nm. Note that the mirrored single image appears at Z = 14.75 mm and the direct single image appears at Z = 28.5 mm, respectively.

In order to verify the theoretical analysis above, simulation based on beam propagation method (BPM) is performed. In the simulation model, the input and output SMFs lengths are set to be 1 mm and the HCCW length is 27.5 mm. The dispersion of material RI is taken into account in the simulation. When the incident wavelength is 1551 nm, a mirrored single image can be formed at a propagation distance of Z = 14.75 mm and a direct single image reproduced from the incident light field at the first splicing joint (Z = 1 mm) can be observed at the second splicing joint (Z = 28.5 mm), as can be seen in Figure 2. Simulation results also show that, when incident wavelength decreases, the imaging distance of the mirrored single image and direct single image will increase, and vice versa. Thus, the image at Z = 28.5 mm disappears gradually when the incident wavelengths deviate from 1551 nm, which means that a peak around 1551 nm in the transmission spectrum of the proposed device can be obtained.

**Figure 3:**
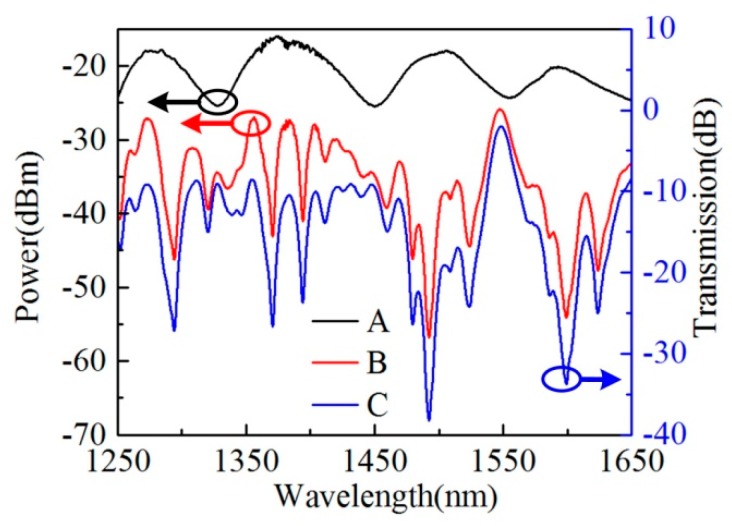
Transmitted power of the light source (curve A) and light power passing through a 27.50-mm-long sample (curve B), and the normalized transmission spectrum of the sample in dB scale (curve C).

In the experiment, a sample with HCCW length of 27.5 mm is fabricated, and the discharge parameters applied in the splicing process are pre-fusion power of standard −40 bit, pre-fusion time of 60 ms, overlap of 9 μm, fusion power of standard −5 bit, and fusion time of 600 ms, respectively, and the transmission spectrum is measured by the OSA in real-time. As shown in Figure 3, curve A and B are the power of the light source and the transmitted power after the sample is inserted, respectively, in dBm, and curve C is the normalized transmission spectrum (subtraction of trace B and trace A) of the sample in dB scale. The large insertion loss of the sample is mainly due to the mismatch of the mode fields and numerical apertures between the HCCW and SMFs. Actually, similar results can be found in many previously reported works [20,21,22,23]. A peak located at 1548.60 nm can be clearly seen in curve C of Figure 3, which corresponds to the self-imaging wavelength of the device. The peak wavelength is slightly different from the simulated one, which is possibly because of the length accuracy of the fabricated sample. In order to show the self-imaging peak more clearly, the sample transmission are shown in linear scale hereafter.

**Figure 4:**
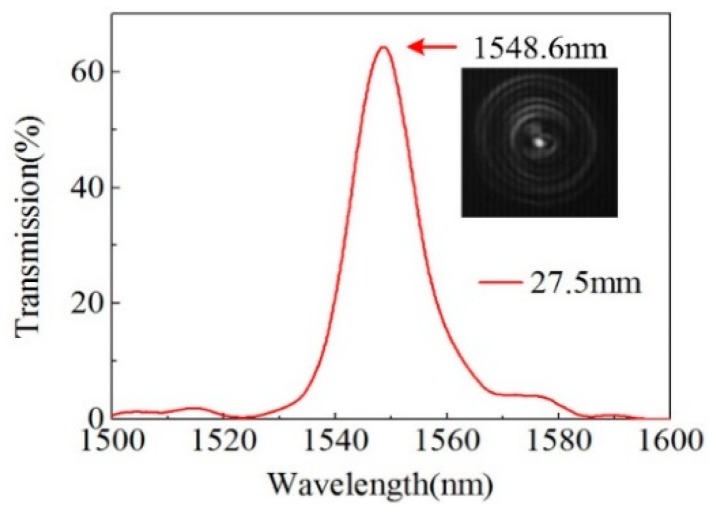
Transmission spectrum of the 27.50-mm-long sample in linear scale. Inset, near-field pattern of the direct single image at the output splicing joint of the sample.

Figure 4 shows the transmission spectrum of the 27.5-mm-long sample in linear scale, where the self-imaging wavelength located at 1548.60 nm can be seen more clearly compared to that shown in Figure 3. To observe the direct single image formed from self-imaging effect, the near-field light pattern at the end of the HCCW is measured with a tunable laser (81940 A, Agilent, Santa Clara, CA, USA) and an infrared charge-coupled device (CCD, model 7290 A, Electro Physics Corp, Fairfield, NJ, USA). The sample abovementioned is cleaved at the second splicing joint, with the input SMF connected to the tunable laser operated at 1548.60 nm with a power of 4 mW and the cleaved end positioned to the focal plane of the infrared CCD. The near field light pattern recorded by the infrared CCD is given as an inset in Figure 4, where we can see that there is a central bright spot in the image, which confirms the direct single image at the second splicing joint (Z = 28.5 mm) as shown in Figure 2 and is in good agreement with the simulation prediction.

**Figure 5:**
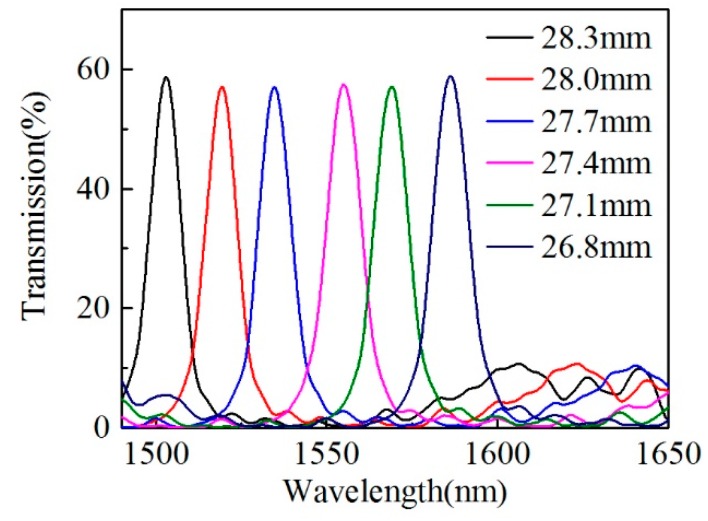
Transmission spectra of samples with HCCW lengths from 28.3 mm to 26.8 mm, where the peak wavelength shift to longer wavelengths with the HCCW length decreases.

According to the simulation results, the self-imaging peak wavelength will shift to longer wavelengths once the HCCW length reduces. The relationship between the peak wavelength and the HCCW length is investigated by fabricating samples with different lengths. Firstly, a sample with HCCW length of 28.3 mm is fabricated and the corresponding transmission spectrum is plotted in Figure 5. Then the HCCW of the sample is cut back by a step of about 0.3 mm for five times, and each time the cut HCCW is fusion spliced with output SMFs again to record the transmission spectrum, which is also plotted in Figure 5 for comparison. The cutting of the HCCW is achieved by employing a standard fiber cleaver (FC-6S, Sumitomo, Tokyo, Japan) under the monitoring of a CCD camera (BC2000, BoCheng, Shenzhen, China), with a cutting precision of <10 μm. As can be seen in Figure 5, the peak wavelengths shift from 1502.95 nm to 1586.29 nm when HCCW length of the samples reduce from 28.3 mm to 26.8 mm, which is in good agreement with Equation (1) and our simulation predictions. Note that all the fusion splicing parameters are kept the same for all these samples.

**Figure 6:**
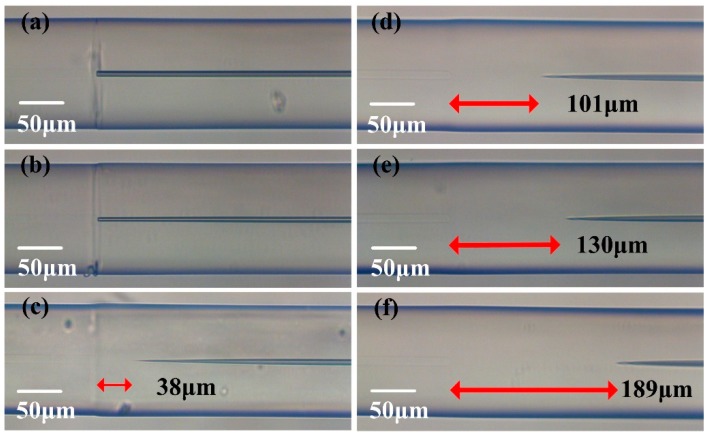
(**a**~**f**) Microscope image of the splicing joints of six sample with fusion time of 200 ms, 400 ms, 600 ms, 800 ms, 1000 ms and 2000 ms, respectively.

**Figure 7:**
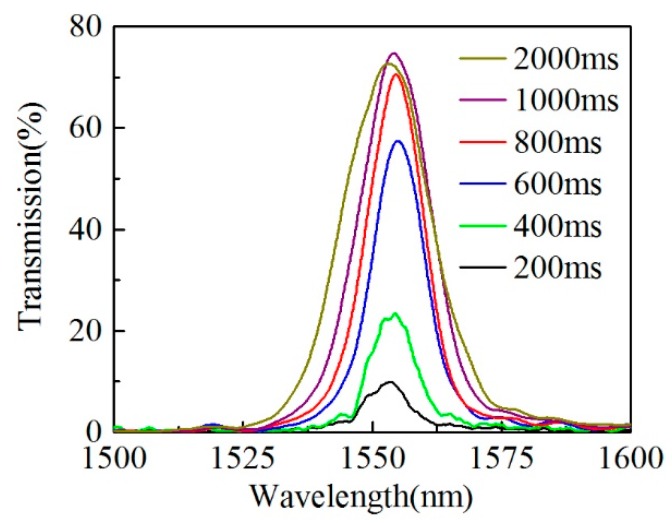
Transmission spectra of six samples with same HCCW length (27.4 mm) but different fusion time.

The collapse effect of the HCCW during fusion splicing is also investigated since it affects the actual HCCW length of the samples and thus the light coupling conditions at the splicing joints. Here, the arc fusion parameters are kept the same as mentioned above, except for the fusion time is set to be 200 ms, 400 ms, 600 ms, 800 ms, 1000 ms and 2000 ms, respectively, to fabricate six samples with similar HCCW lengths (~27.4 mm) but different collapse lengths. The microscope images of the splicing joints of these samples are shown in Figure 6. When the fusion time is set as 200 ms and 400 ms, the collapse of HCCW can be ignored, as can be seen in Figure 6a,b. The air hole of HCCW starts to collapse when fusion time exceeds 600 ms, and the collapsing length of HCCW increases apparently from 38 μm to 189 μm with fusion time increasing from 600 ms to 2000 ms. And the transmission spectra of these samples are plotted in Figure 7, where we can see that the collapse of the air hole actually improves the light coupling efficiency at around the splicing joint. This is possibly because that more power of the self-imaging wavelength can be coupled into the HCCW when there is collapse region at the splicing joints. In addition, the optimized fusion time can be estimated to be 1000 ms according to the transmission spectra shown in Figure 7.

## 3. Sensing Characteristics

Benefited from the operation principle of self-imaging effect, the proposed structure can be used for RI and temperature sensing. With considering its embedded hollow core structure, its sensing performance may be further enhanced through liquid infiltration. Therefore, two samples with HCCW length of 30.2 mm, one of which is filled with index matching liquid (Cargille Labs, Cedar Grove, NJ, USA) in the air hole of the HCCW, are fabricated for RI and temperature measurement. The RI of the liquid used is 1.446 at 589.3 nm (1.435 at 1548.6 nm) when room temperature is 25 °C, which is slightly lower than that of silica. For the fabrication of the liquid-filled sample, a section of HCCW is filled with index matching liquid through capillary effect and then cut to the expected length, and finally fusion spliced to SMFs at both ends. To avoid harmful effects induced by liquid evaporation and carbonization during fusion splicing, pre-cleaning discharge with reduced discharge time and power is performed before the normal fusion splicing to achieve an optimized splice loss. Due to liquid evaporation, the liquid-length in HCCW of the filled sample is 25.6 mm, and two hollow voids in adjacent of the two splicing joints remains, with lengths of 3.2 mm and 1.4 mm, respectively.

**Figure 8:**
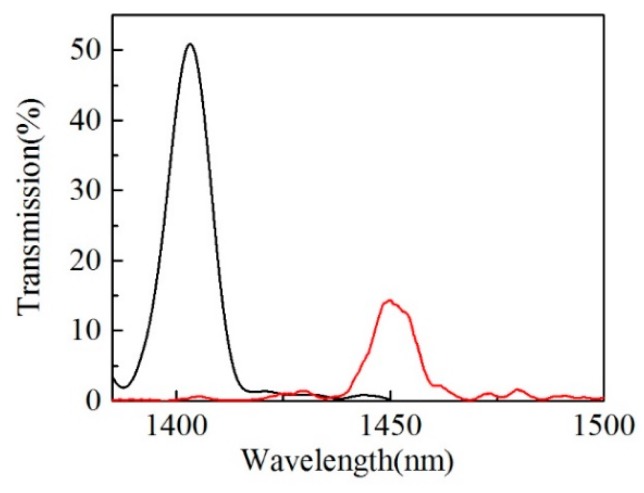
Transmission spectra of the un-filled sample (black curve) and liquid-filled sample (red curve) at 25 °C. HCCW length of the samples are 30.2 mm.

Transmission spectra of the un-filled and liquid-filled samples abovementioned are plotted in Figure 8, where we can see that the peak wavelength of the un-filled and liquid-filled samples are 1403.15 nm and 1449.94 nm, respectively. It indicates that the introduction of liquid to the air hole of HCCW brings red shift of the peak wavelength. The red shift of peak wavelength is mainly caused by the liquid induced effective RI increasing and mode field expansion of the HCCW [15].

**Figure 9:**
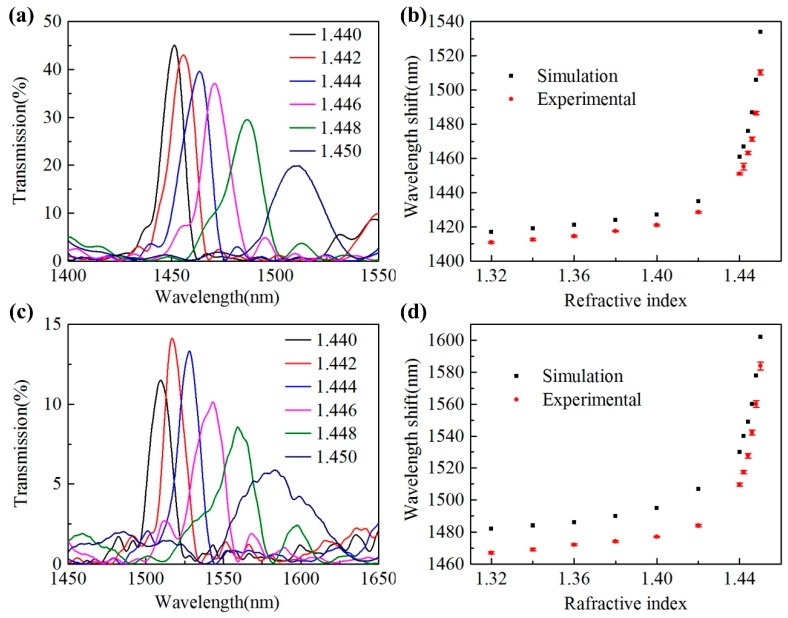
(**a**) Transmission spectra of the un-filled sample under different refractive indexes (RIs). (**b**) Experimental (red circles) and simulated (black squares) relationship between the peak wavelength and surrounding RI of the un-filled sample. (**c**) Transmission spectra of the liquid-filled sample under different RIs. (**d**) Experimental (red circles) and simulated (black squares) relationship between the peak wavelength and surrounding RI of the liquid-filled sample.

To investigate the device response to surrounding RI, the two samples are immersed into a series of standard index matching liquids, respectively. During the RI test, the spectrum is swept and recorded by OSA for 10 times for each RI test to obtain an averaged peak wavelength, and then the sample is cleaned carefully with alcohol after each test until the original spectrum restored. Figure 9 shows the transmission spectra of the un-filled and liquid-filled sample under different RIs and the relationship between their peak wavelength and surrounding RI. For both cases, the peak wavelength shifts to longer wavelengths with RI increasing. And owing to the working principle of the proposed device, self-imaging phenomenon generated by multimode interference, the RI response shows obvious nonlinearity when surrounding RI is approaching to the material RI of HCCW for both samples. This is resulted from the effective modal RI increasing and mode field expansion in case of surrounding RI increasing [15], however, an analytical, or even semi-analytical expressions for the nonlinearity of RI response is difficult to be derived due to the intrinsic complexity of the multimode-guidance nature of the HCCW. Therefore, BPM simulations have been conducted to better understand this nonlinearity, and the simulation results are plotted in Figure 9b,d as black squares for comparison with the experimental results. Overall, both the simulation and experimental results show similar nonlinear wavelength shift and they agrees well with each other, despite there are slight discrepancies of peak wavelengths between the simulation and the experiment, which can be attributed to the HCCW length differences between the simulation model and the actual samples.

To evaluate the experimental obtained RI sensitivities, averaged sensitivities between 1.32–1.44 and 1.440–1.450 for both samples are calculated and listed in Table 1. For the un-filled sample, the peak wavelength shifts from 1410.87 nm to 1451.04 nm in the RI range of 1.32–1.44 (averaged RI sensitivity of 335 nm/RIU), and then shifts from 1451.04 nm to 1510.27 nm for RI increasing from 1.440 to 1.450 (averaged RI sensitivity of 5923 nm/RIU). Especially, considering a smaller RI range from 1.448 to 1.450, the peak wavelength shift is 23.84 nm, which corresponds to an RI sensitivity of 11920 nm/RIU. Accordingly, the averaged RI sensitivities of the liquid-filled sample in 1.32–1.44 and 1.440–1.450 are estimated to be 355 nm/RIU (peak wavelength shift from 1467.01 nm to 1509.58 nm) and 7433 nm/RIU (peak wavelength shift from 1509.58 nm to 1583.91 nm), respectively. In addition, the RI sensitivity for the liquid-filled sample achieves 12,005 nm/RIU in 1.448–1.450, with a peak wavelength shift of 27.77 nm.

Detection limit (DL) is another important parameter for evaluating the sensor performance. According to the heuristic formula given by White and Fan [24], DL can be evaluated by DL = R/S, with taking into account the signal-to-noise ratio (SNR), full width at half-maximum (FWHM) value of the resonance, thermal noise, and the OSA resolution, where R is the minimum detectable change in response wavelength and S is the sensitivity of the sensor. For both samples, FWHM is the main factor that determines the DL of the proposed device. Assuming a spectral SNR of 50 dB and ignoring the thermal noise, DL calculated according to aforementioned sensitivities of both samples at different RI ranges are also listed in Table 1. The DL is on the order of 10^−3^ RIU and 10^−4^ RIU in the RI range of 1.32–1.44 and 1.440–1.450, respectively, for both samples, which are better or comparable to that of previously reported works that employing MMF-CSF-MMF, SMF-CSF, SMF-HCF couplers or SMF-MMF-SMF structure [11,13,25,26], as listed in Table 2.

**Figure 10:**
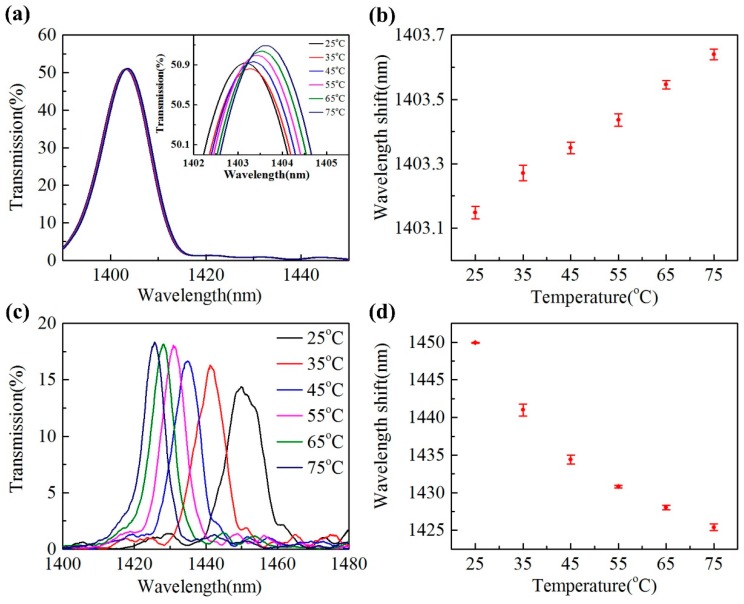
(**a**) Transmission spectra of the un-filled sample under various temperatures. (**b**) Peak wavelength of the un-filled versus temperature. (**c**) Transmission spectra of the liquid-filled sample under various temperatures. (**d**) Peak wavelength of the liquid-filled sample versus temperature.

For temperature sensing, both samples are placed in a high precision column oven (LCO 102, Ecom, Chrastany, Czech Republic) with an accuracy of ±0.1 °C to test their temperature response. The temperature is increased from 25 °C to 75 °C gradually with an interval of 10 °C, at each temperature the samples are maintained for 15 min. Transmission spectra and peak wavelengths for both samples at different temperature are shown in Figure 10. Here, the spectrum is also swept for 10 times to obtain an average peak wavelength.

For the un-filled sample, the peak wavelength shifts from 1403.15 nm to 1403.64 nm when temperature increases from 25 °C to 75 °C, which gives an average temperature sensitivity of 9.8 pm/°C. The top right inset of Figure 10a shows the enlarged spectra around the peak wavelength of the un-filled sample for clarity. The wavelength shift of the un-filled sample is mainly caused by thermal-optic effect of silica, which can be clearly explained by Equation (2) that derived directly from Equation (1),

(2) $$\frac{d\lambda }{dT}=\frac{8D^{2}}{L}\frac{dn}{dT}=\frac{\lambda }{n}\frac{dn}{dT}$$

here *D* is 60 μm and *L* is 30.2 mm. With ignoring the HCCW expansion in diameter and length and taking into account the thermo-optic coefficient of silica (1.1 × 10^−5^/°C) according to reference [27], the temperature sensitivity of un-filled sample can be calculated to be 10.5 pm/°C, which agrees well with the experimental results.

On the other hand, the liquid-filled sample exhibits blue shift in peak wavelength when temperature is increased from 25 °C to 75 °C, as shown in Figure 10c,d. With temperature increasing from 25 °C to 75 °C, the average peak wavelength shifts from 1449.94 nm to 1425.40 nm, exhibiting an averaged sensitivity of −0.49 nm/°C. Particularly, the sensitivity achieves −0.89 nm/°C in the temperature range of 25–35 °C, where the average peak wavelength shifts from 1449.94 nm to 1441.00 nm. Apparently, the temperature sensitivity of the liquid-filled sample is enhanced nearly 50 times compared to that of the un-filled sample. The high temperature sensitivity is mainly attributed to the high thermal-optic coefficient (−3.96 × 10^−4^/°C) of the index matching liquid, which affect the self-imaging wavelength efficiently through introducing large surrounding RI change of the HCCW during the temperature test. Of course, the proposed sensor exhibits limited sensitivity in temperature and RI sensing compared to the reported ultra-sensitive structures, such as that of employing waveguide coupling [4,5]. However, it is still meaningful to introduce the self-imaging effect in a HCCW into the fields of fiber optic sensing, because more fiber sensors and devices, especially with the integration of optical functional materials, can be expected to be designed and demonstrated with improved performances and novel applications.

## 4. Conclusions

In conclusion, a fiber-optic sensor based on self-imaging effect in HCCW is demonstrated for sensing applications. The device is mainly composed of a section of HCCW that sandwiched between two SMFs. The self-imaging effect is investigated with different fiber lengths and arc-fusion parameters. The sensing performance of the sensor is experimentally investigated with a liquid-infiltrated structure and an un-filled one. The temperature sensitivity of the device can be improved significantly with the infiltrated structure and reaches −0.49 nm/°C when temperature is increased from 25 °C to 75 °C. Both the un-filled and liquid-filled device exhibit approximately the same RI sensitivity of ~12,000 nm/RIU in the RI range of 1.448–1.450. The proposed sensor is simple, highly sensitive and cost effective, which may find potential applications in physical and chemical sensing.

## Figures and Tables

**Table 1:** Averaged refractive index (RI) sensitivity and detection limit (DL) of the un-filled and liquid-filled samples.

	Un-Filled Sample		Liquid-Filled Sample	
RI Range	RI Sensitivity (nm/RIU)	DL (RIU)	RI Sensitivity (nm/RIU)	DL (RIU)
1.32–1.44	335	1.34 × 10^−3^	355	1.73 × 10^−3^
1.440–1.450	5923	1.76 × 10^−4^	7433	2.69 × 10^−4^

**Table 2:** Sensitivity and DL comparisons of the proposed sensor with previously reported works.

Sensor Type	Range	Sensitivity	DL
MMF-CSF-MMF [11]	1.30~1.44	229 nm/RIU	4.37 × 10^−4^
SMF-CSF [13]	1.33~1.38	141 nm/RIU	2.8 × 10^−5^
SMF-HCF coupler [25]	1.331~1.403	4.03 V/RIU	3.5 × 10^−3^
SMF-MMF-SMF [26]	1.431~1.437	1815 nm/RIU	-
This study	1.32~1.44	335 nm/RIU	1.34 × 10^−3^
